# Effect of mobile-phone messaging on patient and health-worker knowledge and adherence to the isoniazid preventive therapy guideline in HIV clinics in Southeast, Nigeria

**DOI:** 10.1186/s12879-021-06759-4

**Published:** 2021-10-19

**Authors:** Ifeyinwa Chizoba Akamike, Ijeoma Nkem Okedo-Alex, Chihurumnanya Alo, Adaoha Pearl Agu, Chigozie Jesse Uneke, Lawrence Ulu Ogbonnaya

**Affiliations:** 1 Department of Community Medicine, Alex Ekwueme Federal University Teaching Hospital, Abakaliki, Ebonyi Nigeria; 2 Department of Community Medicine, Ebonyi State University, Abakaliki, Ebonyi Nigeria; 3 African Institute for Health Policy and Health Systems, Ebonyi State University, Abakaliki, Nigeria

**Keywords:** Mobile phone messaging, Knowledge, Adherence, Isoniazid Preventive Therapy, Guideline

## Abstract

**Background:**

HIV-infected persons are at increased risk of developing tuberculosis and Isoniazid preventive therapy has been shown to reduce the occurrence of tuberculosis among this group of persons. M-health technology has been reported to increase both knowledge and implementation of various health services including Isoniazid preventive therapy implementation. This study aimed to determine the effect of m-health on health worker knowledge and adherence to isoniazid preventive therapy (IPT) guidelines and on patient knowledge and adherence to isoniazid treatment.

**Methods:**

This was a quasi-experimental study that was carried out in six health facilities in Ebonyi State, southeast Nigeria. Three health facilities were assigned to each arm (intervention and control arms) and all eligible health workers (total population of 45 and 41 in intervention and control arms respectively) were recruited. Data were also collected from 200 patients (100 per arm). The intervention consisted of mobile phone messages and reminders for health workers on the IPT guideline. Chi-square test was carried out at p < 0.05 and 95% confidence interval.

**Results:**

At baseline, 54.5% and 63.4% of health workers in intervention and control arms respectively had good knowledge which improved significantly to 90.2% in the intervention arm after the intervention (*χ*^*2*^ = 14.22, p < 0.0001). At baseline, 61.4% and 90.2% of health workers had good adherence to the guideline in intervention and control arms respectively which also improved in the intervention arm by 28.8% after intervention although not significant(*χ*^*2*^ = 0.37, p = 0.54). More than 50% of the patients in both study arms had poor knowledge, with the intervention arm having a significantly higher proportion of respondents (68.0%) with poor knowledge at baseline (*χ*^*2*^ = 4.71, p = 0.03). The proportion of patients with good knowledge however increased significantly (88.8%) in the intervention arm after intervention (*χ*^*2*^ = 25.65, p < 0.001). Patients had good adherence to IPT in intervention and control arms before (100% and 84.2% respectively) and after (96.6% and 100% respectively) the study. There was no significant difference in adherence among patients in both arms.

**Conclusions:**

Health worker knowledge and practice of guidelines as well as patient knowledge improved in the intervention arm in this study. These findings suggest the consideration for the inclusion of mobile phone reminders in the guideline for tuberculosis prevention among HIV patients.

## Introduction

Tuberculosis (TB), an opportunistic infection in Human immunodeficiency virus (HIV) disease is worsening in areas with a high prevalence of HIV [1]. By the end of the first year of HIV infection, there is a doubling of the risk of active TB which worsens with a decrease in the CD4 lymphocyte [1]. People living with HIV/AIDS are about 20 to 37 times more likely to develop TB than those who are not HIV infected [2, 3]. The bidirectional relationship between HIV and TB affects the prognosis of patients and further complicates clinical diagnosis and treatment plans [2]. As a result of this interaction between TB and HIV, integration of TB and HIV services is recommended [4]. The integration of TB and HIV services leads to better services for clients and much more impact on TB prevention. One of the first steps in achieving this integration is the provision of isoniazid at the same clinic where people receive their ART [4]. The use of IPT is one of the approaches for ending the TB epidemic by 2030 which is one of the core targets of the Sustainable Development Goal three.

The World Health Organization (WHO) and the joint United Nations Programme on HIV and AIDS (UNAIDS) issued a policy statement in 1999 recognising the effectiveness of preventive therapy for TB in people living with HIV/AIDS (PLWHA) [1, 5]. A recommendation was made for the use of targeted isoniazid preventive therapy (IPT) as part of the package of care for PLWHA [5]. The steps to be taken according to the recommended guidelines included (1) counseling on TB; (2) screening for active TB; (3) targeting of those most likely to benefit from IPT; (4) provision of IPT to those without active TB; (5) monitoring for adherence and toxicity of IPT; and (6) evaluation of the outcome of TB [5].

Despite these recommendations, studies have reported low rates of IPT coverage. Studies in western countries showed that only 47–80% of physicians provided IPT for their HIV-infected patients according to the guideline [5]. Lower coverage has been reported in Africa. A study in Addis Ababa revealed that only 32% of TB-free PLWHA were provided with IPT [6] while a similar study carried out in Ethiopia showed IPT coverage to range from 8.1% to 47% [7]. Initiation of Isoniazid preventive therapy requires patient motivation and understanding of the diagnosis and also adequate communication between health workers and the patient [8]. These factors affect adherence. Even if patients are initiated on IPT, the benefit is only realized if patients complete their prescribed therapy [9]. Studies have reported that adherence rates for Isoniazid Preventive Therapy vary significantly from 34% to 98% [3, 8, 10–13].

To scale up services in developing countries, adherence to treatment adherence is a very important factor that should be considered [2]. Adherence is an active, deliberate, and responsible process of care whereby the patient works to maintain health in collaboration with healthcare providers [14].

The role of physicians and other health workers in improving adherence through proper education of the patients on the importance of IPT cannot be over-emphasized [10]. Studies have shown that individuals who were educated on the need to take IPT by their doctors/nurses are more likely to adhere to therapy than those who were not counselled [15, 16]. It is also important for health workers to adhere to the IPT guidelines, this will help in the effective implementation of the IPT program among HIV patients [17]. A study carried out in Thailand showed that, although more than half of studied physicians had seen the WHO guidelines for providing IPT in HIV-infected patients, the adherence to this guideline was still low [5].

M-health is the use of mobile phone technology for health-related purposes. It involves the use and capitalization on mobile phone applications such as voice calls and short messaging services (SMS) in disseminating health information [18, 19]. Mobile health has become widespread, particularly in developing countries [20]. Studies have shown the effectiveness of m-health technology in improving health outcomes for various chronic diseases including HIV [21–24]. A systematic review provided evidence on behavior change and clinical outcomes from randomized or quasi-experimental controlled trials of text message interventions. The review showed that of nine sufficiently powered studies, eight found evidence to support text messaging as a tool for behavior change [25].

A good number of healthcare workers have cell phones and the provision of information through repeated texting is a strategy that can be used to improve healthcare worker’s knowledge about IPT and IPT guidelines [26]. Weekly reminders using mobile phone texting could also be of benefit in improving healthcare worker adherence to IPT guidelines and thereby improve patient knowledge and adherence to IPT [26]. Therefore this study set out to determine the effect of mobile phone messaging for health workers on knowledge and adherence to the isoniazid preventive therapy guideline among health workers and on patient knowledge and adherence to isoniazid preventive therapy in HIV clinics in Ebonyi State, Nigeria.

## Methods

### Study setting

The study was carried out in six health facilities providing comprehensive care for HIV patients in Ebonyi State. Ebonyi State is located in the Southeastern part of the country. The health service delivery system in Ebonyi State is structured into a three-tier system with the primary health care at the base, supported by the secondary and tertiary health care levels. The state has two tertiary health facilities, several registered public and private health facilities including 13 general hospitals, six mission hospitals, and a plethora of primary health care centres and private hospitals/clinics. Public, private and mission hospitals provide comprehensive HIV care in the state. Comprehensive HIV care includes voluntary and confidential counselling and testing, prevention of HIV transmission, including mother-to-child transmission, prophylaxis against opportunistic infections, diagnosis and treatment of HIV-related conditions including opportunistic infections, and antiretroviral treatment [27].

### Study design and population

A quasi-experimental study comprising of an intervention arm and a control arm was carried out among health care workers who render services at the HIV clinics and among people living with HIV who access HIV care at the selected health facilities.

### Sample size determination

For the health workers, the sample size formula for comparing two proportions at a significance level of 5% and desired power of 80%,p1 (i.e. health worker adherence to guideline after the intervention; taken as 81%) and p2 (health worker adherence to guideline without intervention; taken as 51%) [28], with an attrition rate of 20% was used to arrive at a sample size of 44. A sample of 85 health workers, 44 participants from the intervention group and 41 from the control group were recruited. Only 41 participants were eligible to participate in the control arm.

The sample size calculation for health workers is shown below:

The formula for comparing two dependent proportions is:

$$\frac{{n = \left( {Z\alpha + Z\beta } \right)^{2} \times (P1\left( {1 - P1} \right) + P2\left( {1 - P2} \right)}}{{\left( {P1 - P2} \right)^{2} }}$$

where

n = minimum sample size in each group.

Zα = 1.96, the critical ratio or standard normal deviate at significance level of 5%

Zβ = 0.84, the critical ratio or standard normal deviate at desired power of 80%.

P_1_ = health worker adherence to guideline after intervention; taken as 81%

P_2_ = health worker adherence to guideline without intervention; taken as 51%

Inserting the required information in the formula:

$$\frac{{n = \left( {1.96 + 0.84} \right)^{2} \times (0.81\left( {1 - 0.81} \right) + 0.51\left( {1 - 0.51} \right)}}{{\left( {0.81 - 0.51} \right)^{2} }}$$

*n* = 35.

To compensate for loss to follow up, an adjustment was made to the calculated sample size. Using a response rate of 80%, the sample size was calculated by dividing the originally calculated size (n) by the anticipated response rate i.e. n/0.8 = 35/0.8 which gave 44 approximately.

For the patients, a total of 200 patients were selected for this study. The formula for comparing two proportions was used considering a significance level of 5%, desired power of 80%, p1(patient adherence rate to therapy after the intervention of 76.9%), and p2 (patient adherence rate to therapy without intervention taken as 55.8%), with an attrition rate of 20% to arrive at 93. Data were collected from 100 respondents for each arm (intervention and control) of the quasi-experimental study. These details of sample size determination for the PLWHA have been reported in a previous paper [29].

The sample size calculation for the HIV patients is shown below

The formula for comparing two dependent proportions is:

$$\frac{{n = \left( {Z\alpha + Z\beta } \right)^{2} \times (P1\left( {1 - P1} \right) + P2\left( {1 - P2} \right)}}{{\left( {P1 - P2} \right)^{2} }}$$

where

n = minimum sample size in each group.

Zα = 1.96, the critical ratio or standard normal deviate at significance level of 5%

Zβ = 0.84, the critical ratio or standard normal deviate at desired power of 80%.

P_1_ = patient adherence rate to therapy after intervention of 76.9%

P_2_ = patient adherence rate to therapy without intervention taken as 55.8%

Inserting the required information in the formula:

$$\frac{{n = \left( {1.96 + 0.84} \right)^{2} X(0.769 \left( {1 - 0.769} \right) + 0.558\left( {1 - 0.558} \right)}}{{\left( {0.769 - 0.558} \right)^{2} }}$$

*n*=74

To compensate for loss to follow up, an adjustment was made to the calculated sample size. Using an anticipated response rate of 80%, the sample size was calculated by dividing the originally calculated size (n) by the anticipated response rate i.e. n/0.8 = 74/0.8 which gave 93 approximately.

### Sampling technique

Facility sampling was restricted to only facilities recording more than 100 HIV patients. Using a simple random sampling technique, six health facilities were selected from a list of the health facilities that provide comprehensive HIV care in the State. These facilities were first grouped into public and private health facilities. The public health facilities were assigned as the intervention arm while the private/mission hospitals were assigned as the control arm. All the eligible health workers working in the selected clinics were included in the study. For the patients, proportionate allocation of sample size was done to ascertain the sample size per health facility. Most of the health facilities run at least one clinic day a week and patients are given at most two monthly appointments, therefore data was collected for 2 months and all eligible patients were selected till the sample size was reached. These details of the sampling technique for the PLWHA have been described in a previous paper [29].

### Data collection

The study was in three phases namely baseline phase, intervention phase, and post-intervention phase. For the health workers, a pre-tested structured self-administered questionnaire was used to collect information on socio-demographic characteristics of the respondents, their knowledge of guidelines for IPT, healthcare worker practice of prescribing IPT, and educating the patient on same.

For the PLWHA a pre-tested structured interviewer-administered questionnaire was used to collect information on the socio-demographic characteristics of the respondents, their knowledge about IPT, and patient adherence to IPT. The content validity of the questionnaires for this study is based on recent guidelines for IPT for PLWHA, the literature reviewed, and experts' feedback. In addition, both the content and face validity was checked during the pre-test and also by the experts in the related field. Interviews were carried out by trained research assistants.

Baseline data collection was carried out in 2018 in the HIV clinics of the selected health facilities and lasted 2 months.

### Intervention component

The intervention was directed at the health workers in the intervention arm and consisted of mobile phone messaging using SMS to provide information on the isoniazid preventive therapy guideline to the health workers and also as reminders for them to implement the guideline. The SMS was sent weekly because most of the health facilities run at least one clinic every week. The content of the text messages included: eligibility criteria, steps to be taken in commencing IPT, the dosage of isoniazid for IPT according to the national guideline for isoniazid preventive therapy. The SMS was sent on the morning of the clinic days. Provision of information using SMS lasted for 3 months. The SMS was aimed at improving the adherence of health workers as regards the IPT guideline. The intervention lasted for 3 months. At the end of these 3 months, post-intervention data collection was carried out to determine the effect of the intervention on health worker knowledge and adherence to Isoniazid preventive therapy guidelines and on patient knowledge and adherence to Isoniazid preventive therapy.

### Data management and statistical analysis

#### Health worker variables

The independent variables were socio-demographic and work characteristics of health workers such as age, gender, marital status, professional level, duration of service in ART clinic. Dependent variables were health worker knowledge of IPT and health worker adherence to IPT guideline. Nine questions assessed the knowledge of healthcare providers on IPT eligibility criteria, IPT provision, and its management. A correct answer for each close- ended question was given 1 score and 0 score was given for a wrong answer. The score varied from 0 to 9 points and was classified into 2 levels (Good: 5–9 scores (≥ 55%), and Poor: 0–< 5 scores (< 55%)). Five questions were used to assess health worker's adherence to the guideline. The five questions ask how often the healthcare providers provide IPT for PLWHA and monitor the treatment in conformity to the IPT guideline. The questions had three choices (Yes always which was given a score of two, Yes sometimes which was scored one, and No which was given zero). The total score varied from 0 to 10, and was classified into two levels (Good: 5–10 scores (≥ 50%), and Poor: 0– < 5 scores (< 50%).

### Patient variables

The independent variables were socio-demographic characteristics of patients such as age, gender, marital status, educational level, and employment status.

Five questions were used to assess knowledge of PLWHA about isoniazid preventive therapy. A correct answer for each was given a score of 2, and 0 was given for a wrong answer. The score varied from 0–10 points and was classified into two levels (Good: 5–10 scores (≥ 50%), and Poor: 0– < 5 scores (< 50%) [29], while Patient Adherence to IPT was measured by self-reported adherence to IPT in the last 30 days.

Data were analysed using Statistical Package for Social Sciences (IBM-SPSS) for Microsoft Windows version 20. The level of significance was set at p < 0.05 and the confidence interval at 95%. The chi-square test was carried out to test for the effect of the intervention on knowledge and adherence.

## Results

### Health worker results

Table 1 shows that a higher proportion of the respondents in the control arm were less than 30 years. A significantly higher proportion of the respondents in the intervention group were married when compared to the control group (*χ*^*2*^ = 6.31, p < 0.01). Also, a significantly higher proportion of the respondents in the control arm were “other health workers” (Nurses, Pharmacists, CHEWS) (*χ*^*2*^ = 11.52, p < 0.001). The majority of the respondents in both arms had worked in the HIV clinic for 5 years and below and a majority had been trained on TB/HIV activities and had ever read the guideline. However, less than 50% had been trained on IPT specifically. Table 2 compares the proportion of health workers that answered the knowledge questions correctly in the intervention and control arms at the beginning and end of the study. At the beginning of the study, a significantly higher proportion of the respondents in the intervention arm answered the question on eligibility for IPT correctly (*χ*^*2*^ = 9.11, p = 0.003). Also, a significantly higher proportion of respondents in the intervention arm knew that chest radiography is not a requirement for screening (*χ*^*2*^ = 10.47, p = 0.001). However, a significantly higher proportion in the control arm knew that IPT was used as secondary prophylaxis for people with a past history of tuberculosis (*χ*^*2*^ = 9.50, p = 0.002).

**Table 1:** Socio-demographic and work characteristics of health workers

Variable	Intervention	Control	*χ*^*2*^	*p *value
	N = 44 n (%)	N = 41 n (%)		
Age group (years)				
< 30	2 (4.5)	15 (36.6)	16.86	< 0.001^a^
30–39	17 (38.6)	15 (36.6)		
≥ 40	25 (56.8)	11 (26.8)		
Mean age (mean ± SD)	40.43 ± 7.16	33.15 ± 8.56	4.26^b^	< 0.001^a^
Gender				
Male	19 (43.2)	17 (41.5)	0.03	0.87
Female	25 (56.8)	24 (58.5)		
Marital status				
Married	34 (77.3)	21 (51.2)	6.31	0.01^a^
Others^c^	10 (22.7)	20 (48.8)		
Professional level				
Medical Doctor	23 (52.3)	7 (17.1)	11.52	0.001^a^
Other health workers^d^	21 (47.7)	34 (82.9)		
Duration of work in HIV clinic				
6–11 months	7 (15.9)	12 (29.3)	2.24	0.52
1–3 years	13 (29.5)	11 (26.8)		
> 3 years–5 years	11 (25.0)	8 (19.5)		
> 5 years	13 (29.5)	10 (24.4)		
Training on TB/HIV activities				
Yes	30 (68.2)	23 (56.1)	1.32	0.25
No	14 (31.8)	18 (43.9)		
Training on IPT				
Yes	15 (34.1)	17 (41.5)	0.49	0.48
No	29 (65.9)	24 (58.5)		
Ever read guideline				
Yes	27 (61.4)	25 (61.0)	0.001	0.97
No	17 (38.9)	16 (39.0)		

^a^Statistical significance ^b^T test

^c^Single, divorced, widowed, separated

^d^Nurses, Pharmacists, CHEWS

**Table 2:** Comparison of intervention and control arms based on knowledge questions answered by health workers at the beginning and end of study

Knowledge question	Beginning of study				End of study			
	Intervention arm	Control arm	*χ*^*2*^	*p *value	Intervention arm	Control arm	*χ*^*2*^	*p *value
	N = 44 n (%)	N = 41 n (%)			N = 41 n (%)	N = 35 n (%)		
IPT reduces the risk of TB infection for HIV positive patients	42 (95.5)	39 (95.1)	FT	1.0	41 (100.0)	33 (94.3)	FT	0.21
Patients with active TB should not be given IPT	33 (75.0)	34 (82.9)	0.80	0.37	41 (100.0)	28 (80.0)	FT	0.003^a^
Knowledge of TB symptoms	37 (84.1)	39 (95.1)	FT	0.2	37 (90.2)	31 (88.6)	FT	1.0
Is chest radiography a requirement for screening	26 (59.1)	10 (24.4)	10.47	0.001^a^	26 (63.4)	6 (17.1)	16.58	< 0.001^a^
Knowledge about eligibility for IPT	15 (34.1)	3 (7.3)	9.11	0.003^a^	29 (70.7)	1 (2.9)	36.41	< 0.001^a^
Is current pregnancy a contraindication for starting IPT	31 (70.5)	32 (78.0)	0.64	0.42	36 (87.8)	29 (82.9)	0.37	0.54
IPT as secondary prophylaxis for people with past history of TB	24 (54.5)	35 (85.4)	9.50	0.002^a^	33 (80.5)	30 (85.7)	036	0.55
Best TB preventive treatment drug	40 (90.9)	37 (90.2)	FT	1.0	41 (100.0)	30 (85.7)	FT	0.02
Isoniazid drug dose	35 (79.5)	34 (82.9)	0.16	0.79	41 (100.0)	34 (97.1)	FT	0.46
Overall Knowledge score								
Good	24 (54.5)	26 (63.4)	0.69	0.40	37 (90.2)	18 (51.4)	14.22	< 0.001^a^
Poor	20 (45.5)	15 (36.6)			4 (9.8)	17 (48.6)		

^a^Statistical significance FT-Fisher exact

At the end of the study, a significantly higher proportion of health workers in the intervention arm knew that IPT should not be given to patients with active TB (FT, p < 0.003). For the questions on IPT eligibility and chest radiography as a requirement for screening, significantly higher proportions (*χ*^*2*^ = 36.41, P < 0.001 and *χ*^*2*^ = 16.58, p < 0.001 respectively) of respondents in the intervention arm answered the questions correctly when compared to the control arm. For the question on the use of IPT as secondary prophylaxis for people with a past history of TB, the statistical significance that was present at the beginning of the study disappeared with an increase in the proportion of health workers in the intervention arm answering the question correctly, though the proportion was still slightly lower than that of the control group. For overall knowledge, over 50% of the respondents in the intervention and control arms of the study had good knowledge at the beginning of the study. At the end of the study, a significantly higher proportion of respondents in the intervention arm had good knowledge when compared to the control arm (*χ*^*2*^ = 14.22, p < 0.001).

Table 3 shows that at the beginning of the study, the majority of the health workers in both arms adhered to the guideline. For all the adherence items, a majority of the respondents (> 70%) affirmed that they adhered to the guideline. However, a significantly higher proportion of health workers in the control arm answered yes to the question on the provision of isoniazid for eligible patients (FT, p = 0.003). After the intervention, the proportion of respondents that answered yes to the adherence questions in the intervention arm was higher for all the practice questions when compared to the control arm, however, these differences were not statistically significant. For the question on the provision of isoniazid for eligible patients, the statistical significance disappeared after the intervention and the intervention arm now had a higher proportion answering yes to this question. For overall practice, at the beginning of the study, the proportion of health workers with good adherence was 28.8% higher in the control arm when compared with the intervention arm. This difference was statistically significant (*χ*^*2*^ = 9.52, p = 0.002). However, after the intervention, there was an increase in the good adherence proportion to 90.2% in the intervention arm with a difference of 4.5% when compared with the control arm. This difference in the proportion of good adherence between intervention and control arms post-intervention was not statistically significant (*χ*^*2*^ = 0.54, p = 0.37).

**Table 3:** Comparison of proportion of health workers practicing the IPT guideline correctly in the intervention and control arms at the beginning and end of the study

Adherence Item	Beginning of Study				End of Study			
	Intervention arm	Control arm	*χ*^*2*^	*p *value	Intervention arm	Control arm	*χ*^*2*^	*p *value
	N = 44 n (%)	N = 41 n (%)			N = 41 n (%)	N = 35 n (%)		
Use of TB screening tool (algorithm) to identify PLWHA eligible for IPT	34 (77.3)	38 (92.7)	3.89	0.05	36 (87.8)	74.3 (26)	2.30	0.13
Encourage PLWHA to start IPT once they are eligible	40 (90.9)	37 (90.2)	FT	1.0	40 (97.5)	32 (91.4)	FT	0.33
Provide INH for eligible HIV+ patients	35 (79.5)	41 (100.0)	FT	0.003^a^	38 (92.7)	32 (91.4)	FT	1.0
Educate clients on IPT & on the need to adhere to their treatment	41 (93.2)	38 (92.6)	FT	1.0	41 (100.0)	32 (91.5)	FT	0.09
Monitor and manage clients with INH drug toxicity	32 (72.8)	36 (87.8)	3.02	0.08	36 (87.8)	30 (85.7)	0.07	0.79
Overall Adherence score								
Good	27 (61.4)	37 (90.2)	9.52	< 0.002^a^	37 (90.2)	30 (85.7)	0.37	0.54^b^
Poor	17 (38.6)	4(9.8)			4 (9.8)	5 (14.3)		

^a^Statistical significance, FT-Fisher exact, ^b^Likelihood ratio

Result tables for Patients

### Patient characteristics

Table 4 shows the socio-demographic characteristics of PLWHAs. The two arms of the study are comparable in their socio-demographic characteristics. The mean age of the respondents was 38.87 ± 9.28 and 40.02 ± 11.21 in the intervention and control arms respectively. The majority of the respondents in both arms were females, married, and were employed. Slightly less than 50% of the respondents in the intervention arm had at most primary education while slightly more than 50% had at most primary education in the control arm. Table 5 shows that at the beginning of the study, the control arm had a significantly higher proportion of patients who answered the questions correctly except for the questions on knowledge of drug name and knowledge of who should receive IPT for which the control group had higher proportions but not statistically significant (*χ*^*2*^ = 1.84, p = 0.18 and *χ*^*2*^ = 3.08, p = 0.21 respectively). After the intervention, the intervention arm had significantly higher proportions of patients answering the knowledge questions correctly except for the question on knowledge of drug name for which the higher proportion in the intervention arm was not statistically significant. Overall, more than 50% of the respondents in both study arms had poor knowledge, with the intervention arm having a significantly higher proportion of respondents with poor knowledge at baseline (*χ*^*2*^ = 4.71, p = 0.03). After the intervention, the proportion with good knowledge increased significantly in the intervention arm (88.8%) when compared with the control arm (54.8%), (*χ*^*2*^ = 25.65, p < 0.001).

**Table 4:** Socio-demographic characteristics of patients

Variable	Intervention arm	Control arm	*χ*^*2*^	*p *value
	N = 100 n (%)	N = 100 n (%)		
Age Group (years)				
< 30	15 (15.0)	18 (18.0)	0.88	0.83
30–39	38 (38.0)	32 (32.0)		
40–49	29 (29.0)	31 (31.0)		
≥ 50	18 (18.0)	19 (19.0)		
Mean age (mean ± SD)	38.87 ± 9.28	40.02 ± 11.21	0.79^a^	0.43
Gender				
Male	27 (27.00)	26 (26.00)	0.03	0.87
Female	73 (73.00)	74 (74.00)		
Marital status				
Married	60 (60.00)	64 (64.00)	0.34	0.56
Others	40 (40.00)	36 (36.00)		
Level of education				
Primary education and less	46 (46.00)	54 (54.00)	1.28	0.26
Secondary education and more	54 (54.00)	46 (46.00)		
Employment status				
Employed	83 (83.00)	73 (73.00)	2.9	0.09
Unemployed	17 (17.00)	27 (27.00)		

^a^t test

**Table 5:** Comparison of patient knowledge and overall knowledge scores between intervention and control arms

Knowledge Item	Beginning of study				End of study			
	Intervention arm n = 100 Correct	Control arm n = 100 Correct			Intervention arm n = 89	Control arm n = 93		
Knowledge of isoniazid function	42 (42%)	63 (63%)	7.88	0.02^a^	82 (92.1)	65 (69.9)	14.49	< 0.001^a^
Knowledge of IPT service availability	41 (41%)	56 (56%)	4.50	0.03^a^	85 (95.5)	58 (62.4)	29.67	< 0.001^a^
Knowledge of drug name for IPT	8 (8%)	14 (14%)	1.84	0.18	29 (32.6)	21 (22.6)	2.29	0.13
Knowledge of duration of treatment	33 (33%)	51 (51%)	6.65	0.02^a^	76 (85.4)	49 (52.7)	22.62	< 0.001^a^
Knowledge of who should receive IPT	21 (21%)	29 (29%)	3.08	0.21	73 (82.0)	36 (38.7)	35.52	< 0.001^a^
Overall Knowledge score								
Good	32 (32.0)	53 (53.0)	4.71	0.03^a^	79 (88.8)	51 (54.8)	25.65	< 0.001^a^
Poor	68 (68.0)	47 (47.0)			10 (11.2)	42 (45.2)		

^a^Statistical significance

Table 6 shows that after the intervention, the proportion of respondents that were on IPT increased to 66.3% and 40.9% from 16 to 19% respectively in the intervention and control arms. The intervention arm had a significantly higher proportion of respondents on IPT when compared with the control arm (*χ*^*2*^ = 11.82, p = 0.001). At the beginning of the study, the adherence level of respondents was very high, 100% and 84.2% in intervention and control groups respectively and there was no statistically significant difference in both arms. After the intervention, there was also no significant difference in adherence level in both arms.

**Table 6:** IPT uptake and patient adherence

	Beginning of study			End of study		
	Intervention	Control	*χ*^*2*^ (*p *value)	Intervention	Control	*χ*^*2*^ (*p *value)
	n = 100 n(%)	n = 100 n (%)		n = 89 n (%)	n = 93 n (%)	
IPT uptake						
Yes	16 (16.0)	19 (19.0)	0.31 (0.58)	59 (66.3)	38 (40.9)	11.82 (0.001*)
No	84 (84.0)	81 (81.0)		30 (33.7)	55 (59.1)	

Adherence grading	n = 16 n (%)	n = 19 n (%)	*χ*^*2*^ (*p *value)	n = 59 n (%)	n = 38 n (%)	*χ*^*2*^ (*p* value)
Good	16 (100.0)	16 (84.2)	FT (0.23)	57 (96.6)	38 (100.0)	FT (0.52)
Poor	0 (0.0)	3 (15.8)		2 (3.4)	0 (0.0)	

*Statistical significance

## Discussion

This study assessed the effect of mobile phone-based education and reminders on health worker knowledge and adherence to isoniazid preventive therapy guideline and patient knowledge and adherence to isoniazid preventive therapy. At the beginning of the study, over 50% of the health workers answered almost all the knowledge questions correctly except the question on eligibility for IPT. The proportion however improved significantly after intervention in the intervention arm. Also, a majority of the health workers in the control arm wrongly reported that chest radiography was a requirement for screening patients for commencement of IPT. This agrees with a study carried out in Ethiopia where more than half of the respondents (55.2%) incorrectly answered the question on the necessity of chest radiography for screening PLWHA for IPT eligibility [30]. According to the WHO IPT guideline, chest radiography is no longer a mandatory investigation before starting IPT i.e. chest radiography can be done if available, but is not required to classify patients into TB and non-TB groups [17]. At post-intervention, the proportion that answered correctly for all the knowledge questions increased with 70% answering correctly the question on IPT eligibility in the intervention arm. Being aware of the groups of patients that are eligible for IPT will lead to easy identification of people who should receive prophylaxis.

At the beginning of the study, a majority of the health workers in both intervention and control arms had good knowledge. This finding agrees with the result of a study carried out in northern Nigeria [31]. This similarity may be because these studies were carried out in the same country where similar guidelines are used, similar service training attended by the health workers, and similar development partners involved in HIV care. The good knowledge is however in contrast with the reports of a systematic review that showed a high level of inappropriate knowledge among health workers [32]. Improvements in knowledge about treatment guideline are important for health workers because it leads to effective implementation of these guidelines and can lead to improvement in clinical outcomes and cost. This is also of great importance in maintaining high quality in the management of HIV [33].

A majority of the health workers in both groups had good adherence to the guideline in both intervention and control groups at the beginning of the study. The control arm however had a significantly higher proportion of good adherence when compared with the intervention arm. This higher proportion could be because the control arm comprised faith-based hospitals. The fact that the intervention arm had public hospitals while the control arm had faith-based hospitals may have affected this finding. However, anecdotal evidence suggests that there is a lot of motivation for workers in faith-based hospitals. In addition, while discussing with clinic heads in both arms during the study, it was gathered that in faith-based hospitals, every worker who attends training, is mandated to step down the training to other colleagues. This is unlike the public or government-owned hospitals where a majority of workers do not take responsibility as a result of poor monitoring and evaluation systems. The good adherence to IPT guidelines seen in this study correlates with previous studies [30, 34–36], but does not agree with a study carried out in Brazil and another in Thailand which showed that adherence of health workers to the IPT guideline was poor [5, 37].

The m-health technology intervention that was employed in this study was found to be useful in improving both knowledge and adherence to the IPT guideline among health workers. The level of knowledge significantly improved among the health workers when compared with the control arm after the intervention. In addition, the proportion of health workers that had good practice was significantly higher in the control arm at the beginning of the study, however, the intervention arm was able to cover this gap and had a higher proportion of good adherence after the intervention. Electronic reminders and monitoring have been used in many disease conditions including HIV [38]. There is a high prevalence of cell phone use globally and the low and middle income countries including Nigeria are not excluded. This provides new opportunities to incorporate mobile phones into health service delivery [26, 38, 39]. The fragile health systems faced with high prevalence of tropical diseases, high rate of infectious diseases, high mortality rate, in low and middle-income countries (LMICs) will benefit from mobile health technologies which have now been recognized as a less expensive and easy means of providing high-quality healthcare services [40]. Integration of mobile health into health service provision in LMICs could reach more people in resource-limited settings than the traditional forms of disease control.

Concerning knowledge of IPT among patients, although both groups had more than 50% of respondents having poor knowledge at baseline, the respondents in the control arm had more information about IPT. Improvements in patient knowledge are valuable because knowledge about the disease and medications prescribed, an understanding of the benefits of the medication, and good attitudes toward treatment, all contribute to better medication-taking behavior and are associated with higher rates of adherence [41].

Although the intervention was not directed at the patients, the health workers who received the short messages were encouraged to educate the patients on IPT, prescribe IPT for patients and also educate them on the importance of adherence. There was a 6.8% increase in the proportion of health workers that educated their patients although this increase was not significant. Patient education plays a significant role in improving both knowledge and patient outcome. The study also showed that the proportion of patients receiving IPT increased significantly within both study arms after the intervention, however, the intervention arm had higher proportion of patients receiving IPT when compared with the control arm. This implies that the intervention may have contributed in increasing uptake of IPT in this study. Patient adherence rate was good in both intervention and control arms both at the beginning and end of the study. There was no significant difference in adherence between the intervention and control arms both before the intervention and at the end of the study. This good adherence to therapy is particularly important in achieving the agenda of the sustainable development target of ending TB by 2030.

M-health technology has been reported to increase both knowledge and implementation of various health services including Isoniazid preventive therapy implementation [22, 38, 42, 43]. Mobile text messaging strategy of m-health technology used in this study had a unique effect in addressing some of the barriers to implementation of IPT and in improving knowledge. Information on the IPT guideline was sent by text messages in bits weekly over a period of 3 months. This served to improve the knowledge the health workers had on the guideline. Short messages are easy to read and also easy to assimilate. An added advantage of short messages is that the receiver can decide to read the message at a convenient time. In addition to the information provided, short messages that served as reminders to the health workers were sent weekly. This helped to create more consciousness in the health workers and served as a motivation for them to implement the guideline.

One limitation of this study is the small sample size of health workers which is because the study was carried out in clinics specific for HIV care with only a few health workers. A second limitation is also the self-reporting of adherence to guidelines by health workers and adherence to therapy by patients. Additionally, there’s a possibility of the study participants receiving information from other clinicians, although there was no similar intervention or program ongoing at the time of the study. Another concern is the fact that allocation was based on public versus private/mission hospital. However, this allocation was based on the nature of the HIV clinics. These clinics are comparable since both the public and private/mission provide comprehensive HIV care and the clinic composition and activities are similar. This is because HIV care is provided by donor agencies in these health facilities and so similar routines are obtainable in the two arms. In addition, each arm had a specialist/referral facility and two secondary health facilities. The authors also admit the possibility that text messages although received may not have been read. However, researchers carried out on-site visits and asked respondents if they received and read text messages. Respondents were asked to contact the principal investigator if text messages were not received, however, none did so. Also, a few of the participants were called to confirm receipt of the messages. One of the strengths of this study is that intervention included mobile phone SMS which the respondents could read at their own convenient time. Again, this study assessed both health worker and patient outcomes of the topic and so gives a comprehensive view of the subject. Additionally, m-health is an important tool that can be useful in improving health and so this study provides evidence for the use of m-health technology in HIV/TB control.

## Conclusion

In this study, m-health technology was shown to be effective in improving knowledge and adherence to IPT guidelines among health workers and in improving knowledge among patients. We recommend that program implementers should consider including text message reminders as part of the routine in TB/HIV activities. This will help increase the consciousness of health workers towards IPT implementation. Frequent training to improve health workers’ knowledge on the updated recommendations of IPT guidelines are essential in keeping the health workers informed of any guideline changes.

## Data Availability

The datasets used and/or analyzed during the current study are available from the corresponding author on reasonable request.

## Abbreviations

IPT: Isoniazid Preventive Therapy
TB: Tuberculosis
HIV: Human Immunodeficiency Virus
SMS: Short messaging service
PLWHA: People living with HIV/AIDS
WHO: World Health Organisation
ART: Antiretroviral therapy
UNAIDS: United Nations Programme on HIV and AIDS
FT: Fisher exact test

## References

[1] Alarcon E, Bissell K, Boillot F, Caminero JA, Chiang C, Clevenbergh P (2015). Isoniazid preventive therapy for people living with HIV : public health challenges and implementation issues. Int J Tuberc Lung Dis.

[2] Makanjuola T, Taddese HB, Booth A (2014). Factors associated with adherence to treatment with isoniazid for the prevention of tuberculosis amongst people living with HIV/AIDS: a systematic review of qualitative data. PLoS ONE.

[3] Getachew Y, Mekonnen W (2015). Correlates of adherence and utilization of Isoniazid preventive therapy in HIV patients. J Microbiol Infect Dis.

[4] Isoniazid Preventive Therapy (IPT) for the Prevention of Tuberculosis in People Living with HIV (e-course)|FHI 360. https://www.fhi360.org/resource/isoniazid-preventive-therapy-ipt-prevention-tuberculosis-people-living-hiv-e-course.

[5] Hiransuthikul N, Hiransuthikul P, Nelson KE, Jirawisit M, Paewplot R, Kasak S (2005). Physician adherence to isoniazid preventive therapy guidelines for HIV infected patients in Thailand. Southeast Asian J Trop Med Public Heal.

[6] Wesen A, Mitike G (2012). Provision and awareness for isoniazid preventive therapy among PLHIV in Addis Ababa, Ethiopia. BMC Int Health Hum Rights.

[7] Teklay G, Teklu T, Legesse B, Tedla K, Klinkenberg E (2016). Barriers in the implementation of isoniazid preventive therapy for people living with HIV in Northern Ethiopia: a mixed quantitative and qualitative study. BMC Public Health.

[8] Goswami ND, Gadkowski LB, Piedrahita C, Bissette D, Ahearn MA, Blain MLM (2012). Predictors of latent tuberculosis treatment initiation and completion at a U. S. public health clinic : a prospective cohort study. BMC Public Health.

[9] Adams LV, Talbot EA, Odato K, Blunt H, Steingart KR (2014). Interventions to improve delivery of isoniazid preventive therapy: an overview of systematic reviews. BMC Infect Dis.

[10] De ADS, Paula A (2015). Factors associated with treatment for latent tuberculosis in persons living with HIV / AIDS Fatores associados ao tratamento da tuberculose latente em pessoas vivendo com HIV/AIDS Factores asociados al tratamiento de la tuberculosis latente en personas. Cad saúde pública.

[11] Ngamvithayapong J, Uthaivoravit W, Yanai H, Akarasewi P, Sawanpanyalert P (1996). Adherence to tuberculosis preventive therapy among HIV-infected persons in Chiang Rai, Thailand. AIDS.

[12] Yamin A, Bornstein E, Hensel R, Mohamed O, Kempker RR. Predictors of latent tuberculosis infection treatment after introduction of a new regimen : a retrospective cohort study at an inner city clinic. Open Forum Infect Dis. 2016;(Cdc):1–6. 10.1093/ofid/ofw082 PMC5066457 27757409

[13] Shayo GA, Moshiro C, Aboud S, Bakari M, Mugusi FM (2015). Acceptability and adherence to Isoniazid preventive therapy in HIV-infected patients clinically screened for latent tuberculosis in Dar es Salaam, Tanzania. BMC Infect Dis.

[14] Schneider MP, Krummenacher I, Figueiredo H, Marquis J, Bugnon O (2009). Adherence: a review of education, research, practice and policy in Switzerland. Pharm Pract (Granada).

[15] Berhe M, Demissie M, Tesfaye G (2014). Isoniazid preventive therapy adherence and associated factors among HIV positive patients in Addis Ababa, Ethiopia. Adv Epiemiol..

[16] Mindachew M, Deribew A, Tessema F, Biadgilign S, Smoll M, Shafer F (2011). Predictors of adherence to isoniazid preventive therapy among HIV positive adults in Addis Ababa, Ethiopia. BMC Public Health.

[17] Federal Ministry of Health Department of public health. Guideline for isoniazid preventive therapy. 2014.

[18] World Health Organisation (2011). mHealth: new horizons for health through mobile technologies. Glob Obs eHealth Ser.

[19] Betjeman TJ, Soghoian SE, Foran MP (2013). mHealth in Sub-Saharan Africa. Int J Telemed Appl.

[20] Lau YK, Cassidy T, Hacking D, Brittain K, Haricharan HJ, Heap M (2014). Antenatal health promotion via short message service at a Midwife Obstetrics Unit in South Africa: a mixed methods study. BMC Pregnancy Childbirth.

[21] Richman AR, Maddy L, Torres E, Goldberg EJ (2016). A randomized intervention study to evaluate whether electronic messaging can increase human papillomavirus vaccine completion and knowledge among college students. J Am Coll Heal.

[22] Lee HY, Koopmeiners JS, McHugh J, Raveis VH, Ahluwalia JS (2016). mhealth pilot study: text messaging intervention to promote HPV vaccination. Am J Health Behav.

[23] Schnall R (2015). Short message service use in clinical care through a simulation activity. J Nurs Educ.

[24] Maduka O, Tobin-West CI (2013). Adherence counseling and reminder text messages improve uptake of antiretroviral therapy in a tertiary hospital in Nigeria. Niger J Clin Pract.

[25] Heather C-L, Kershaw T (2010). Text messaging as a tool for behavior change in disease prevention and management. Epidemiol Rev.

[26] Nglazi MD, Bekker L, Wood R, Hussey GD, Wiysonge CS (2013). Mobile phone text messaging for promoting adherence to anti-tuberculosis treatment : a systematic review. BMC Infect Dis.

[27] Kitahata MM, Tegger MK, Wagner EH, Holmes KK (2002). Comprehensive health care for people infected with HIV in developing countries. BMJ.

[28] Zaeh S, Kempker R, Stenehjem E, Blumberg HM, Temesgen O, Ofotokun I (2013). Improving tuberculosis screening and isoniazid preventive therapy in an HIV clinic in Addis Ababa, Ethiopia. Int J Tuberc Lung Dis.

[29] Akamike IC, Okedo-Alex IN, Agu AP, Alo C, Ogbonnaya LU (2020). Knowledge and adherence to isoniazid preventive therapy among people living with HIV (PLHIV) in multilevel health facilities in South-East, Nigeria: baseline findings from a quasi-experimental study. Pan Afr Med J.

[30] Tikuye AM, Tikuye AM. Knowledge, attitudes and practices of health care providers towards isoniazide preventive therapy (IPT) provision in Addis Ababa, Ethiopia; 2013.

[31] Lawan BM, Agu KA (2018). Assessment of short term Isoniazid Preventive Therapy ( IPT ) in people living with HIV/AIDS. West Afr J Pharm.

[32] Manissero D, Marieke J, Langendam M, Huitric E (2012). Knowledge of tuberculosis-treatment prescription of health workers: a systematic review. Eur Respir J.

[33] Akamike IC, Okedo-alex IN, Uneke CJ, Uro-chukwu C, Chukwu OE, Ugwu NI (2020). Evaluation of the effect of an educational intervention on knowledge and adherence to HIV guidelines among frontline health workers in Alex-Ekwueme Federal University Teaching Hospital Abakaliki, Nigeria. Afr Health Sci.

[34] Abdulrazaak AT, Govender I, Nzaumvila D, Group F. Knowledge , attitudes and practices of doctors regarding isoniazid preventive therapy in HIV/AIDS patients at Odi District Hospital, Gauteng province, South Africa Knowledge , attitudes and practices of doctors regarding isoniazid preventive therapy i. South African J Infect Dis. 2018;0053.

[35] Denegetu AW, Dolamo BL (2014). Tuberculosis case finding and isoniazid preventive therapy among people living with HIV at public health facilities of Addis Ababa, Ethiopia : a cross-sectional facility based study. BMC Public Health.

[36] Ngombo AM. Factors affecting implementation of isoniazid preventive therapy in HIV/AIDS clinics in Dar es Salaam Region, Tanzania. 2013;

[37] Saraceni V, Antonio G, Jonathan G, Victoria V, Bonnie K, Solange C (2011). Physician adherence to guidelines for tuberculosis and HIV care in Rio de Janeiro, Brazil. Brazilian J Infect Dis.

[38] Liu X, Lewis JJ, Zhang H, Lu W, Zhang S, Zheng G (2015). Effectiveness of electronic reminders to improve medication adherence in tuberculosis patients: a cluster-randomised. PLoS Med.

[39] National Telehealth Technology Assessment Resource Center. mHealth - Technology Overview. www.telehealthtechnology.org/toolkits/mhealth/about-this-technology/technology-overview.

[40] World Health Organization. Global diffusion of eHealth, Making universal health coverage achievable: report on the Third Global Survey on eHealth Geneva. 2016.

[41] Awwad O, Akour A, Al-Muhaissen S, Morisky D (2015). The influence of patients’ knowledge on adherence to their chronic medications: a cross-sectional study in Jordan. Int J Clin Pharm.

[42] Liu Q, Abba K, Mm A, Sinclair D, Vm B, Mad L (2014). Reminder systems to improve patient adherence to tuberculosis clinic appointments for diagnosis and treatment (Review). Cochrane Database.

[43] Mbuagbaw L, Mursleen S, Lytvyn L, Smieja M, Dolovich L, Thabane L (2015). Mobile phone text messaging interventions for HIV and other chronic diseases: an overview of systematic reviews and framework for evidence transfer. BMC Health Serv Res.

